# Society of Biology

**Published:** 1879-04

**Authors:** O. C. Oliver


### ARTICLE XIV.

SOCIETY OF BIOLOGY. President, M. Paul Bert. Meeting, February 15, 1879. EMPLOYMENT OF PROTOXIDE OF NITROGEN IN SURGICAL OPERATIONS.

M. Paul Bert said all the world knew the use that dentists made of the protoxide of nitrogen to anæsthetize their patients. They employed the protoxide chemically pure. American surgeons have also tried the protoxide of nitrogen in the larger surgical operations, but as it is necessary often to suspend the inhalation in order to avoid danger of asphyxia, and as consciousness returns almost immediately upon withdrawal of the anæsthetic, it follows that continuous operation with this anæsthetic is impossible. The speaker had long sought an anæsthetic at once innocuous and efficacious, and had communicated to the society the results of his experiments on the physiological effects of certain anæsthetics, among them the protoxide of nitrogen. Without detailing his experiments at length, he wished to state that he had been led to give the preference to a mixture of the protoxide with oxygen. The mixture he had employed consisted of 85 parts of the former with 15 of the latter. He found that a bird would live in this mixture for 24 hours. By repeated experiments he had so familiarized himself with the physiological action of this mixture that he tried it in an operation for "ingrowing nail." The pressure in the inhaling chamber was 17 centimeters. The patient, a young woman of 20, slept tranquilly, without having passed through any stage of excitation beyond very few slight contractions.

Almost immediately, on the withdrawal of the anæsthetic the patient recovered consciousness, and was able at once to walk unassisted to the ward, where she partook of her usual meal without any feeling of nausea or other unpleasant sensation except the pain of the wound.

*Lymphatics of the Uterus.*—Dr. Mierzejewski, of St. Petersburgh, presented, through M. Pouchat, a communication on the distribution of the lymphatics of the uterus. He stated that there are two planes of lymphatics; one superficial, the other deep, situated under the peritoneal covering, which two sets of vessels communicate by very free anastomoses. The blood vessels, less abundant than lymphatics, are contrary to the statements of Fridolin and Leopold, situated between the layers of lymphatics. These results are in accord with those obtained by Hoggan.

*Reproduction of the Vitreous Humor.*—M. Phillipeaux had made numerous experiments upon animals which seemed to prove that the vitreous humor is reproduced in the living animal. Punctures of the eye failed to extract entirely the vitreous humor, a small portion always remaining. O. C. OLIVER.

